# A comparative ^18^F-FDG PET/CT imaging of experimental *Staphylococcus aureus* osteomyelitis and *Staphylococcus epidermidis* foreign-body-associated infection in the rabbit tibia

**DOI:** 10.1186/2191-219X-2-41

**Published:** 2012-07-23

**Authors:** Petteri Lankinen, Kaisa Lehtimäki, Antti J Hakanen, Anne Roivainen, Hannu T Aro

**Affiliations:** 1 Orthopaedic Research Unit, Department of Orthopaedic Surgery and Traumatology, University of Turku, Kiinamyllynkatu 10, Turku FI-20520, Finland; 2 Antimicrobial Resistance Unit, National Institute for Health and Welfare, Kiinamyllynkatu 13, Turku FI-20520, Finland; 3 Department of Medical Microbiology and Immunology, University of Turku, Kiinamyllynkatu 13, Turku FI-20520, Finland; 4 Turku PET Centre, University of Turku and Turku University Hospital, Kiinamyllynkatu 4-8, Turku FI-20521, Finland; 5 Turku Center for Disease Modeling, University of Turku, Kiinamyllynkatu 10, Turku FI-20520, Finland

**Keywords:** FDG-PET/CT, osteomyelitis, *Staphylococcus epidermidis*, *Staphylococcus aureus*, biomaterials

## Abstract

**Background:**

^18^F-FDG-PET imaging has emerged as a promising method in the diagnosis of chronic osteomyelitis commonly due to *Staphylococcus aureus*. The inaccuracy of ^18^ F-FDG-PET in the detection of periprosthetic joint infections may be related to the predominance of low-virulent *S. epidermidis* strains as the causative pathogen. We have compared the^18^F-FDG-PET characteristics of *S. aureus* osteomyelitis and foreign-body-associated *S. epidermidis* infections under standardized laboratory conditions.

**Methods:**

Twenty-two rabbits were randomized into three groups. In group 1, a localized osteomyelitis model induced with a clinical strain of *S. aureus* was applied. In groups 2 and 3, a foreign-body-associated infection model induced with a clinical or laboratory strain of *S. epidermidis* was applied. A small block of bone cement was surgically introduced into the medullary cavity of the proximal tibia followed by peri-implant injection of *S. aureus* (1 × 10^5^ CFU/mL) or one of the two *S. epidermidis* (1 × 10^9^ CFU/mL) strains with an adjunct injection of aqueous sodium morrhuate. In group 1, the cement block was surgically removed at 2 weeks but left in place in groups 2 and 3 in order to mimic foreign-body-associated *S. epidermidis* infections. At 8 weeks, the animals were imaged using ^18^ F-FDG PET/CT. The presence of bacterial infection was confirmed by cultures, and the severity of bone infections was graded by means of radiography, peripheral quantitative CT, and semi-quantitative histology.

**Results:**

The *S. aureus* strain caused constantly culture-positive osteomyelitis. The clinical *S. epidermidis* strain resulted in foreign-body-associated infections, while the laboratory *S. epidermidis* strain (ATCC 35983) induced only occasionally culture-positive infections. There was a correlation (*r* = 0.645; *P* = 0.013) between semi-quantitative score of leukocyte infiltration and the ^18^ F-FDG uptake in animals with positive cultures. Standardized uptake value (SUV) of the infected bones was twofold (*P* < 0.001) in *S. aureus* animals compared with *S. epidermidis* animals, but there was only a trend (*P* = 0.053, ANOVA) in the differences of the corresponding SUV ratios. This was due to the altered ^18^ F-FDG uptake of the contralateral tibias probably reflecting a systemic impact of severe osteomyelitis.

**Conclusion:**

The peri-implant inoculation of *S. epidermidis*, reflecting low virulence of the pathogen and limited leukocyte infiltration, was characterized by low ^18^ F-FDG uptake.

## Background

Deep bone infections are one of the most feared complications in elective orthopedic reconstructive procedures as well as in trauma surgery. The clinical situation is expected to become even worse because of the emergence of methicillin-resistant staphylococcal infections and due to the fast growing population of fragile aged patients with an increased susceptibility to surgical site infections [1].

Staphylococci are gram-positive cocci, which are divided into two groups based on the ability to produce the blood clotting enzyme coagulase. *Staphylococcus aureus* and *S. epidermidis* represent the most commonly found coagulase-positive and coagulase-negative staphylococcal species, respectively [2]. *S. aureus* is still the most frequent causative of hematogenous and posttraumatic chronic osteomyelitis [3]. On the contrary, *S. epidermidis* represents opportunistic microorganisms, which have emerged as the leading nosocomial pathogen of biomaterial-related bone infections [4]. Related to differences in virulence, the outcome of bone implant infections caused by *S. epidermidis* strains is better than those caused by *S. aureus* strains [5].

Clinical manifestations of *S. aureus* and *S. epidermidis* bone infections markedly differ from each other. *S. aureus* infections usually present as classical pyogenic infections and enzyme-mediated progressive bone destruction and apposition of new bone [3], whereas the clinical picture of a typical biomaterial-related *S. epidermidis* infection is usually indolent with vague signs of infection [4]. Robust attachment to implant surfaces and subsequent biofilm formation is an important phenotypic feature of *S. epidermidis*[6,7]. By nature, these infections are highly challenging for diagnostic imaging and microbiologic studies, and they are also highly resistant to treatment [8-11].

Compared with other imaging modalities (CT, MRI, labeled leukocyte imaging, and gallium imaging), ^18^ F-FDG-PET imaging seems to have a role in confirming or excluding the diagnosis of chronic osteomyelitis of peripheral bones [12,13]. In contrast, a recent AAOS guideline gave only a weak recommendation for nuclear imaging, including ^18^ F-FDG-PET, in the diagnosis of periprosthetic implant infections [14,15]. It is unknown if the causative pathogen contributes to the level of ^18^ F-FDG uptake in PET imaging of bone infections, but it is possible that the relative inaccuracy of ^18^ F-FDG-PET imaging in the detection of periprosthetic joint infections is due to the prominence of *S. epidermidis* as the most common pathogen of these infections. In this study, we compared under standardized laboratory conditions the level of ^18^ F-FDG uptake in PET imaging of bone/implant infections due to *S. aureus* or *S. epidermidis*. Our hypothesis was that, reflecting different pathomechanisms and virulence, infections caused by *S. epidermidis* result in lower ^18^ F-FDG uptake than pyogenic *S. aureus* infections.

## Methods

### Animals

Twenty-two skeletally mature male New Zealand white rabbits (Harlan) weighing a mean of 3,190 g (SD, 375 g) were used. Before surgery, the rabbits were acclimated to their new environment. The Ethical Committee of the University of Turku and the Provincial State Office of Western Finland approved the study protocol (registration no. 1668/06). The experiment was carried out in the Central Animal Laboratory of the Institution which is included in the National GLPC-Compliance Program and is managed according to the European Convention for the Protection of Vertebrate Animals used for Experimental and other Scientific Purposes and the Statute 1076/85 §3 and 1360/90 of the Animal Protection Law in Finland and the EU Directive 86/609.

### Experimental design

The animals were randomized into three groups (*n* = 6 to 8) based on the infection model and the inoculated pathogen. In group 1 (localized osteomyelitis model), a clinical strain of *S. aureus* (52/52A/80, donated by Dr. Mader) was applied. In groups 2 and 3 (foreign-body-associated infection model), a laboratory *S. epidermidis* (ATCC 35983) and a clinical strain of *S. epidermidis* (T-54580) were applied, respectively. The T-54580 *S. epidermidis* strain was retrieved from a young male with postoperative infection of a plated proximal tibial fracture at Turku University Hospital. Each animal underwent a two-stage surgery for induction of bone infection in the medullary cavity of the proximal left tibia. The right contralateral tibia served as the intact control. The first-stage surgery involved inoculation of one of the three pathogens with a small block of bone cement. At 2 weeks, the animals underwent second-look surgery for verification of induced local infection. The bone cement block was left in place in animals with *S. epidermidis* inoculum in order to mimic a foreign-body-associated infection, while it was removed in *S. aureus* animals. Quantitative ^18^ F-FDG PET/CT was done at 8 weeks, and the presence of bone infection was studied with bacterial cultures. Bone structural changes caused by infection were evaluated by radiography and peripheral quantitative computed tomography (pQCT). The infected tibias were harvested, and the severity of osteomyelitis was graded by means of semi-quantitative histology.

### Induction of infection (first stage of surgery)

The localized *S. aureus* osteomyelitis model (stage IIIA in Cierny-Mader classification) was modified from that of Koort et al. [16], and the foreign-body-associated infection model was adopted from that of Mayberry-Carson et al. [17]. The animals were sedated by a subcutaneous injection of midazolam. Anesthesia was induced and maintained by subcutaneous injections of fentanyl citrate-fluanisone. Using an anteromedial surgical approach, a cortical bone window (6 mm × 2.7 mm) was drilled into the proximal medial metaphysis of the left tibia using a high-speed trephine drill bit under saline cooling. Bone marrow was removed with saline lavage. A small block of sterile pre-polymerized pre-shaped bone cement (Palacos R-40, Schering-Plough Europe) was transplanted into the medullary canal. The periosteal layer was sutured over the cortical window, and a volume of 0.1 mL inoculum of *S. aureus* (10^5^ CFU/mL) or one of the two strains of *S. epidermidis* (10^9^ CFU/mL) was injected into the medullary space next to the cement block. The selected doses of the staphylococcal strains were different due to the differences of bacterial virulence. The necessary dose for induction of *S. aureus* osteomyelitis is low due to the high virulence of the pathogen. The necessary dose for induction of *S. epidermidis* infection is much higher due to the low virulence of the strain. The animals inoculated with *S. epidermidis* also received a local injection of 0.1 mL of aqueous sodium morrhuate (5 % wt/vol) (Scleromate, Glenwood, Englewood, NJ, 07631, USA) for induction of local bone necrosis and to promote the development of the infectious process. The relatively low inoculation dose of *S. aureus* 52/52A/80 was adopted from our previous experiment [16]. Development of experimental *S. aureus* osteomyelitis does not require an adjunct use of sodium morrhuate. The inoculation dose of *S. epidermidis* strains was selected based on a pilot study (three animals). The 10^9^ CFU/mL dose was found to induce culture positive infection in the model without adverse effects on the animal well-being. The dose was the highest tested, and it also represented the maximum dose of CFU per milliliter producing viable bacteria. The soft tissues were rinsed with sterile saline before wound closure. An intramuscular injection of naloxone was given to reverse the anesthesia. After surgery, the animals received standard postoperative pain medication (4 mg/kg of carprofen) for three postoperative days. Functional activity was not limited within individual cages.

### Debridement (second-look surgery)

Two weeks after the first-stage surgery, the animals underwent debridement surgery. Using the previous surgical approach, the bone defect area was re-exposed. Debridement included reopening of the cortical window. Swab specimens and small bone chips were taken for bacteriological confirmation of the infection. The cement block was removed in animals with *S. aureus* inoculation. *S. aureus* osteomyelitis is known to progress without the presence of the cement block [16,18], and if left in place, it may induce an uncontrollable progress of *S. aureus* infection into fatal sepsis. The cement block was left in place in animals with *S. epidermidis* inoculation because the persistence of culture-positive *S. epidermidis* bone infection requires the presence of a foreign body [17,19]. The soft tissues were rinsed with sterile saline, and the wound was closed in layers. The postsurgical care was identical as for the first-stage procedure.

### ^18^ F-FDG PET/CT

Comparative ^18^ F-FDG PET/CT was performed at 8 weeks (6 weeks after the second-look surgery). ^18^ F-FDG was synthesized with a computer-controlled apparatus according to a method described by Hamacher et al. [20], resulting to a specific radioactivity of >76 GBq/μmol and a radiochemical purity of >98 %. PET/CT imaging was performed with Discovery VCT (General Electric Medical Systems, Milwaukee, WI, USA) operated in three-dimensional mode. The scanner is a combined 64-slice CT and PET with 24 rings of bismuth germanate detectors, which acquires 47 imaging planes with an axial field of view of 15.7 cm. The plane thickness of the PET scanner is 4.7 mm, and the spatial resolution for 3D mode is 5.12 mm in full width at half maximum in the 1-cm offset from the center of the field of view [21]. Dynamic PET imaging consisting of 4 × 5-min frames was started 40 min after the injection of ^18^ F-FDG. Dynamic PET imaging was performed to avoid the potential effect of animal movement during the scanning period. CT was performed before PET using technical parameters as follows: scan mode ‘helical’, helical slice thickness of 3.75 mm, detector coverage of 20 mm, pitch factor of 0.531:1, voltage of 100 kVp, current of 80 mA, rotation time of 1 s, scan FOV ‘large body,’ and display FOV 50. PET images were reconstructed with an ordered subset expectation maximization algorithm, and CT was reconstructed using bone algorithm. CT data were used for attenuation correction and for anatomical reference fused with PET images.

The animals fasted for 4 h prior to tracer injection. For PET/CT imaging, the animals were sedated during the surgical procedure. A mean of 94.4 MBq of ^18^ F-FDG (SD 8.5, range 81 to 111 MBq) was injected into the ear vein. Quantitative analysis of ^18^ F-FDG uptake was performed for the standardized circular region of interest (radius 3.8 mm) of the operated left tibia and the corresponding region of the contralateral intact right tibia. The levels of ^18^ F-FDG accumulation were reported as the mean standardized uptake value (SUV). The mean SUV was calculated as the mean radioactivity of the ROI divided by the relative injected dose of radioactivity expressed per kilogram of body weight. SUV ratios between the infected and intact contralateral tibias were also calculated.

### Digital radiography and pQCT

After PET imaging, each animal underwent pQCT scanning. Under fentanyl-fluanisone sedation, the operated limbs were placed in a holder for standard positioning. Imaging was performed using a Stratec XCT Research M pQCT device with software version 5.20 (Norland Stratec Medizintechnik GmbH, Birkenfeld, Germany). After an initial scout view for positioning, the proximal tibias were imaged with six consecutive cross-sectional images using a slice distance of 0.75 mm. A voxel size of 0.07 × 0.07 × 0.50 mm^3^ was used. pQCT images were analyzed for the presence of osteomyelitic destruction and reactive new bone formation.

After pQCT imaging, the animals were killed with an intravenous administration of sodium pentobarbital. Standard anteroposterior and lateral digital radiographs were taken, and radiographic changes were classified according to the osteomyelitis grading system of Mader and Wilson [22]. Two independent observers evaluated the radiographs, and their consensual interpretation was used for data analysis.

### Quantitative microbiological analyses

The presence of infection was re-confirmed with bacterial cultures at the time of killing. Using sterile techniques, the bone defect area was exposed, and swab cultures were taken from subfascial soft tissues. The proximal tibia was aseptically cross-sectioned using a high-speed circular saw in order to obtain three (proximal, middle, and distal) bone specimens from the site of bone infection. The proximal and middle segments were sent to histology and the distal bone segment for quantitative bacterial culture.

All swab specimens were cultured for 18 to 20 h at 35 °C on blood agar plates. After snap-freezing in liquid nitrogen and homogenization with mortar and pestle, all specimens (bone chips and the distal bone segment) were vortexed in saline for 5 min, and ten serial tenfold dilutions were done in order to determine the CFU of *S. aureus* or *S. epidermidis* per gram of bone. The dilutions were cultured for 18 to 20 h at 35 °C on blood agar plates. The aseptically harvested bone cement blocks were cultured on blood agar and immediately placed in BBL™ Brain Heart Infusion broth (Becton, Dickinson and Company, Sparks, MD, USA) and incubated up to 5 days at 35 °C. The turbidity of broth samples was observed every day, and positive cultures (i.e., opaque tubes) were plated on blood agar plates. The plates were incubated for 18 to 20 h at 35 °C. Negative broth samples (i.e., clear tubes) were cultured similarly after 2 and 5 days of incubation.

The isolated pathogens were identified based on morphology, the Slidex® Staph Plus latex agglutination test and the API-Staph® identification test (bioMérieux, Marcy l’Etoile, France) [23]. *S. aureus* (ATCC 29213) was used as the positive control and *Enterococcus faecalis* (ATCC 29212) as the negative control.

### Semi-quantitative histopathological analysis

The proximal and middle bone specimens were processed for histology. The proximal specimen was fixed in 70 % ethanol, embedded in isobornylmethacrylate (Technovit 1200 VLC, Kulzer, Wehrheim, Germany) and stained with a modified van Gieson method. The middle bone segment was decalcified, embedded in paraffin, and stained with hematoxylin and eosin. Histological changes were classified according to the osteomyelitis scoring system presented by Petty et al. (Table 1) [24]. Two independent observers classified the histological sections, and the results were presented as the averaged score value of their interpretation.

**Table 1:** **Summary of histological classification by Petty et al.**[24]

**Infection grade**	**Periosteal reaction**	**Cortex**	**Medullary canal**
0	Often absent; if present, laminated and limited to 1 to 2 thin layers; eccentric and often related to off-center drill defect	Haversian canals small and repair rate slow; polymorphonuclear leukocytes not in granulation tissue; occasional subperiosteal resorptive pockets, but no polymorphonuclear leukocytes	Quick repair with woven bone; inflammatory cells range from none to foci of many intact polymorphonuclear leukocytes; these leukocytes associated with macrophage clean-up; diffuse process
1	Usually laminated with 1 to 2 layers; often eccentric but not related to off-center drill defect	Occasional polymorphonuclear leukocytes in Haversian canals	Subtle, diffuse increase in polymorphonuclear leukocytes; microabscesses present, but hard to find
2	Sunburst type; often nearly circumferential; no apparent cause	Focally enlarged Haversian canals filled with granulation tissue and fragmented polymorphonuclear leukocytes; occasional microabscess	Definite diffuse increase in polymorphonuclear leukocytes with fragmented forms; several definite microabscesses
3	Florid, always sunburst type; circumferential	Subperiosteal, endosteal, and intracortical resorption associated with fragmented polymorphonuclear leukocytes; microabscesses	Many microabscesses or great increase in polymorphonuclear leukocytes diffusely
4			As above, but with sinus-tract formation and soft-tissue microabscesses

### Statistical analysis

Data are expressed as mean ± SD. Comparison of ^18^ F-FDG uptake between the *S. aureus* and *S. epidermidis* groups was made by one-way ANOVA with a *post hoc* Tukey-test. Paired *t*-test was applied in the intra-animal comparison of SUVs between the operated and non-operated tibias. Differences in radiographic and histological scores were analyzed using non-parametric Kruskall-Wallis one-way ANOVA on ranks with Dunn’s pairwise comparison test. Comparisons between SUV values and histological scores of leukocyte infiltration were analysed using Spearman correlation test. Statistical analyses were done using SigmaStat 3.0.1 statistical software (Systat Software Inc., Chicago, IL, USA) and SAS 9.3. software (SAS Institute Inc., Cary, NC, USA). A *P* value less than 0.05 was considered significant.

## Results

### Confirmation of staphylococcal infection

In the *S. aureus* group of animals, all bone cultures as well as those of the retrieved cement block were positive for the inoculated clinical strain of *S. aureus* (52/52A/80) (Table 2). Cultures were infrequently positive for the inoculated pathogen in the group of animals with the standard ATCC strain of *S. epidermidis* (Table 2). The clinical *S. epidermidis* strain (T-54580) resulted more consistently in positive bone cultures at 2 weeks, and cultures of cement block were positive for the inoculated *S. epidermidis* in six animals out of eight at 8 weeks (Table 2). The swab cultures taken from subfascial soft tissues were negative for the inoculated pathogens.

**Table 2:** Number of positive bacterial cultures

**Inoculated microorganism**	**At debridement (2 weeks)**		**Final analysis (8 weeks)**	
	**Bone**	**Retrieved cement block**	**Bone**	**Retrieved cement block**
*S. aureus* (52/52A/80)	100 % (6/6)	100 % (6/6)	100 % (6/6)	NA^a^
*S. epidermidis* (ATCC 35983)	25 % (2/8)	NR^b^	0 % (0/8)	25 % (2/8)
*S. epidermidis* (T-54580)	100 % (8/8)	NR^b^	25 % (2/8)	75 % (6/8)

^a^NA, not applicable (removed at 2 weeks); ^b^NR, not removed for analysis.

### Relationship between ^18^ F-FDG-PET uptake, leukocyte infiltration, and inoculated pathogen

**Figure 1:**
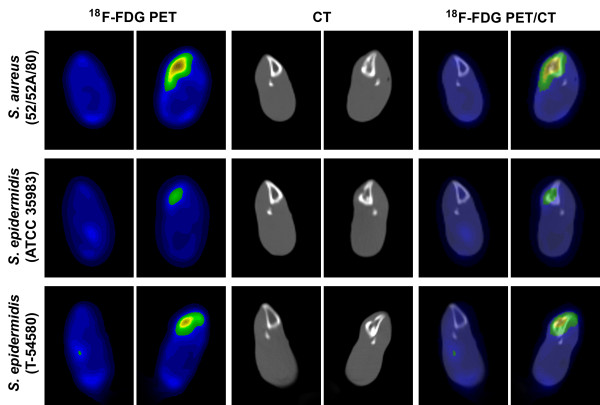
**Transaxial**^**18**^ **F-FDG PET, CT, and combined**^**18**^**F-FDG PET/CT images at the site of induced osteomyelitis.** In the three groups of animals with *S. aureus* (52/52A/80), *S. epidermidis* (ATCC 35983), or *S. epidermidis* (T-54580) inoculum. In each animal, the left tibia (on the right) was infected, and the contralateral intact bone (on the left) served as the control.

PET/CT imaging demonstrated intense accumulation of ^18^ F-FDG in osteomyelitic tibias as compared with the contralateral intact bone (Figure 1). The highest uptake was localized in the medullary cavity. Quantitative analysis of ^18^ F-FDG uptake for the standardized circular region of interest of the infected tibias revealed a relationship between the PET image intensity, the grade of leukocyte infiltration, and the virulence of the inoculated bone pathogen.

There was a significant correlation (*R* = 0.645; *P* = 0.0127) between the semi-quantitative score of leukocyte infiltration in the medullary canal of the tibias and the local ^18^ F-FDG uptake (mean SUV) in animals with positive bacterial cultures of bone and/or retrieved cement blocks (*n* = 14) (*S. aureus* 52/52A/80 *n* = 6, *S. epidermidis* ATCC 35983 *n* = 2, and *S. epidermidis* T-54580 *n* = 6).

**Figure 2:**
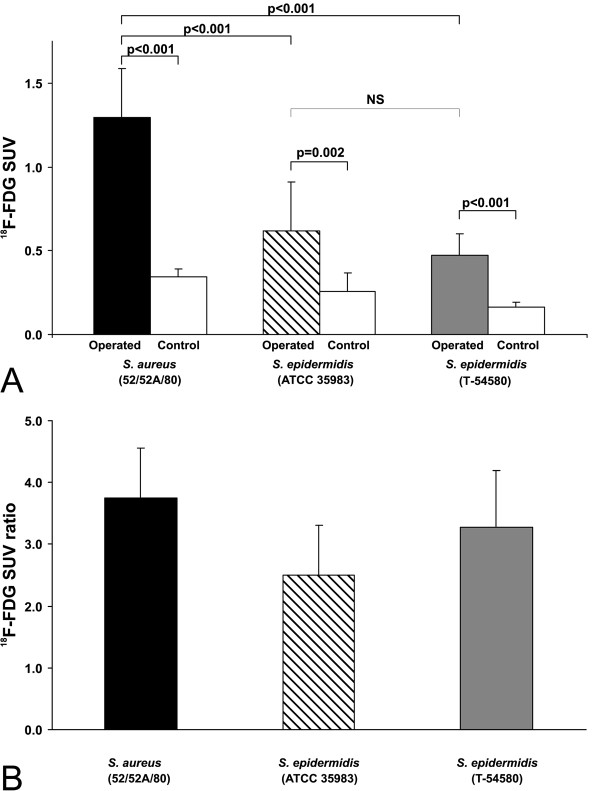
^**18**^**F-FDG PET/CT uptake.** Bar graphs representing SUV (**A**) and SUV ratio (**B**) values (±SD) (*n* = 6 to 8).

The ^18^ F-FDG SUV of the infected bones were significantly higher than those of the contralateral intact tibias in all groups (Figure 2A). The uptake of the infected bones was twofold (*P* < 0.001) in the *S. aureus* group of animals (1.29 ± 0.30) compared with the two *S. epidermidis* (ATCC 35983 and T-54580) groups of animals (0.62 ± 0.30 and 0.47 ± 0.13, respectively)(Figure 2A). The difference of the infected bones in the two groups of animals with *S. epidermidis* inoculation was not statistically significant (*P* = 0.487). The contralateral intact tibias of the *S. epidermidis* animals also showed no significant difference in the uptake. The SUV of the contralateral tibias of the *S. aureus* animals was significantly higher than those of *S. epidermidis* (T-54580) animals (*P* < 0.001), suggesting a systemic impact of staphylococcal osteomyelitis on the physiology of the contralateral limb. As a result the SUV ratios, representing the SUV ratio between the infected and contralateral tibias in each animal showed only a trend (*P* = 0.053, ANOVA) between the animals with *S. aureus*, *S. epidermidis* (ATCC 35983), and *S. epidermidis* (T-54580) inoculations (3.75 ± 0.80, 2.50 ± 0.84, and 2.96 ± 0.98, respectively) (Figure 2B).

### Osteomyelitic changes determined by digital radiography and pQCT

**Figure 3:**
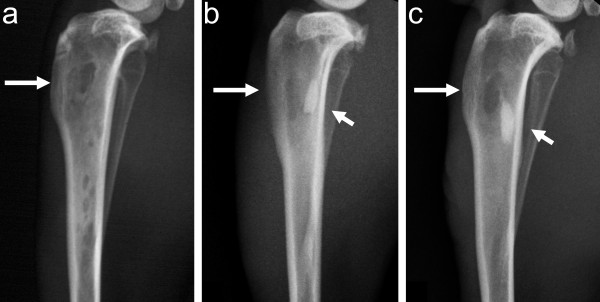
**Lateral radiographs of the infected tibias.** In animals with *S. aureus* (52/52A/80) (**a**), *S. epidermidis* (ATCC 35983) (**b**), or *S. epidermidis* (T-54580) inoculum (**c**) at 8 weeks. The *S. aureus* group of animals (a) showed more prominent periosteal reaction, architectural distortion of the metaphyseal cancellous bone, widening of the tibial shaft, and a completely open cortical window (large arrow). The block of bone cement was visible on the radiographs of the *S. epidermidis* groups of animals (b and c, small arrow). The animals with *S. epidermidis* (ATCC 35983) inoculum (b) showed partial healing of the cortical defect (large arrow) and minimal changes of the local bone architecture. In animals with *S. epidermidis* (T-54580) inoculum (c), the cortical window was open and accompanied with periosteal reaction and minor distortion of the surrounding bone architecture.

On plain radiographs, the group of animals with clinical *S. aureus* (52/52A/80) and *S. epidermidis* (T-54580) strains showed localized osteomyelitis with bone destruction (Figure 3) with similar radiographic scores (3.33 ± 0.82 and 2.57 ± 0.98, respectively). The animals with *S. epidermidis* (ATCC 35983) inoculum showed radiographic signs of cortical bone window healing with low radiological scores (1.19 ± 0.65) (*P <* 0.05 compared with the group of animals with *S. aureus* inoculum).

In pQCT imaging, the group of animals with *S. aureus* (52/52A/80) infection showed cortical bone destruction with circumferential periosteal reaction, reactive endosteal new bone, and sequestrum formation. The animals with *S. epidermidis* (ATCC 35983) inoculum did not exhibit clear osteomyelitic changes on pQCT images, and there were signs of closure in cortical windows. Animals with *S. epidermidis* (T-54580) inoculation showed moderate pQCT signs of local bone infection, including periosteal reaction and small amount of reactive endosteal new bone formation aside with an unhealed cortical window.

### Histological appearance of osteomyelitis

**Figure 4:**
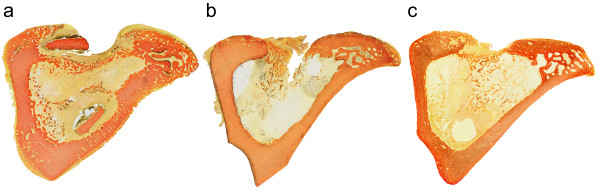
**Cross-sectional histological sections of the infected tibias.** In the animals with *S. aureus* (52/52A/80) (**a**), *S. epidermidis* (ATCC 35983) (**b**), or *S. epidermidis* (T-54580) inoculum (**c**) (van Gieson stains). In animals with *S. aureus* inoculum, drastic osteomyelitic changes is seen characterized by a nearly circumferential periosteal reaction, new bone formation, and sequester formation and infiltration of polymorphonuclear leukocytes with occasional microabscesses. Animals with *S. epidermidis* (ATCC 35983) inoculum did not show signs of infection. In these animals, closure of cortical defect and only a limited number of inflammatory cell infiltrate was seen. In animals with *S. epidermidis* (T-54580) inoculum, signs of chronic/subacute osteomyelitis are seen characterized by periosteal reaction with periosteal sclerosis, infiltration of lymphocytes and plasma cells, and bone marrow showing signs of fibrosis.

The *S. aureus* group of animals showed histologically severe osteomyelitis in all cases (Table 3 and Figure 4) (osteomyelitic score 2.1 ± 0.2). The group of animals with the clinical T-54580 strain of *S. epidermidis* showed signs of chronic or subacute osteomyelitis (osteomyelitis score 1.5 ± 0.3). The *S. epidermidis* ATCC 35983 group of animals showed minimal histological signs of bone infection (osteomyelitis score 0.8 ± 0.4, *P <* 0.05 compared with the *S. aureus* group).

**Table 3:** Average histological scores (±SD) in the study groups

**Inoculated microorganism**	**Periosteal reaction**	**Cortex**	**Medullary canal**	**Osteomyelitis score**
*S. aureus* (52/52A/80)	2.0 ± 0.5	2.1 ± 0.3	2.1 ± 0.3	2.1 ± 0.2
*S. epidermidis* (ATCC 35983)	0.6 ± 0.5	1.0 ± 0.5	0.7 ± 0.5	0.8 ± 0.4
*S. epidermidis* (T-54580)	1.5 ± 0.4	1.5 ± 0.4	1.4 ± 0.6	1.5 ± 0.3

## Discussion

Early diagnosis and treatment of bone implant infections is clinically crucial, but the diagnosis of these infections remains as one of the greatest challenges in the field of orthopedic surgery. The purpose of this study was to determine whether the differences in the virulence of the staphylococcal strains and the severity of subsequent infections contribute to the level of ^18^ F-FDG uptake in PET/CT imaging of osteomyelitis and biomaterial-related bone infection. Indeed, our results suggest that the ^18^ F-FDG uptake of infected bones is related to the degree of leukocyte infiltration, which in turn reflects the virulence of the pathogen.

The histological sections demonstrated the marked difference in the bacterial virulence between *S. aureus* and *S. epidermidis* strains. Classical suppurative osteomyelitis caused by *S. aureus* resulted in an elevated ^18^ F-FDG uptake, while a typical chronic/subacute bone-implant infection caused by the clinical isolate of *S. epidermidis* was characterized by a low uptake of the tracer. Our results explain the reported problems in application of ^18^ F-FDG PET/CT imaging in the diagnosis of periprosthetic implant infections commonly caused by *S. epidermidis*, while the results well agree with the positive results of previous experimental and clinical studies in application of ^18^ F-FDG PET/CT imaging in the diagnosis of osteomyelitis due to *S. aureus*.

Several factors can explain the observed difference in the ^18^ F-FDG accumulation between *S. aureus* and *S. epidermidis* bone infections. It was highly expected that pyogenic *S. aureus* infections with progressive enzyme-mediated bone destruction result on a high local accumulation of ^18^ F-FDG because the tracer uptake is known to be elevated in activated leucocytes, granulocytes, and macrophages. Based on our previous experiments [18], the ^18^ F-FDG uptake can be used even as a predictor of treatment response in a *S. aureus* osteomyelitis. Osteomyelitic tissue destruction is characterized by the presence of concomitant new bone formation. New bone formation *per se* may contribute in the high tracer uptake. Bone healing, involving an inflammatory phase and subsequent new bone formation, is known to mimic infection on PET imaging [16].

The low ^18^ F-FDG tracer uptake in *S. epidermidis* infections was predictable. The virulence of *S. epidermidis* strains is low, and their basic pathomechanism is attachment and slow proliferation within protecting slim (biofilm) on the surface of implants and avascular bone surfaces [4,7]. Within biofilms, *S. epidermidis* bacteria show decreased metabolic activity [25] and marked reduction in glucose uptake [26]. Explaining the clinical picture of indolent infection and treatment resistance, *S. epidermidis* strains are able to survive intracellularly in a quiet state in phagocytic cells and in peri-implant macrophage-like cells [26]. They can invade even into osteoblasts [27,28]. All these features explain the limited inflammatory host response to *S. epidermidis* infections and reflect in the low uptake of the ^18^ F-FDG tracer.

There are no clear explanations for the statistical difference in the ^18^ F-FDG uptake of the contralateral tibias between *S. aureus* animals and those with *S. epidermidis* (T-54580) inoculum. All the groups received the same dose of the tracer, and the imaging procedure has been highly standardized in previous experiments. The phenomenon might be due to the systemic impact of severe staphylococcal osteomyelitis on the physiology of the contralateral limb. It is possible that acute suppurative *S. aureus* osteomyelitis caused a systemic effect exhibiting an increased white blood cell proliferation in the bone marrow of the contralateral tibia. The extent and severity of *S. aureus* osteomyelitis could also have had an effect on the mobility of the animal.

The current experiment demonstrated the low virulence of the selected two *S. epidermidis* strains. Usually by increasing the number of inoculated bacteria, the severity of infection increases. Although we used the maximum applicable *S. epidermidis* concentration (10^9^ CFU/mL), which was high compared to the applied *S. aureus* concentration (10^5^ CFU/mL), the subsequent infection was at best low-grade. It was interesting that the clinically retrieved *S. epidermidis* strain (T-54580) produced in a more reliable manner infection, whereas a constant infection rate could not be achieved with a standard slime-producing *S. epidermidis* strain (ATCC 35983). The finding emphasizes the significance of the inherent properties of the causative *S. epidermidis* strain. The ^18^ F-FDG PET imaging failed to differentiate the two groups of animals inoculated by one of the two *S. epidermidis* strains due to the low uptake in both cases. This is an important observation demonstrating the limited value of the current ^18^ F-FDG PET/CT imaging techniques in the characterization of peri-implant *S. epidermidis* infections.

There are obvious limitations in the clinical application of PET imaging in the diagnostics of skeletal and implant infections. The intensity of the ^18^ F-FDG uptake, as measured by the SUV, is not always a reliable indicator of infection. For example, aseptic loosening of a hip prosthesis may show high ^18^ F-FDG uptake due to the accumulation of inflammatory cells in the periprosthetic soft tissues, mimicking an infection [29].

Our results suggest the relevance to register the causative pathogen in clinical ^18^ F-FDG PET/CT studies. Interestingly, most of the clinical ^18^ F-FDG studies of chronic osteomyelitis and periprosthetic infections have not reported the causative pathogens. The results have rather been presented as culture positive or negative cases. Therefore, it is difficult to draw any definitive conclusions based on the current clinical literature.

### Limitations

The applied rabbit model of *S. aureus* osteomyelitis simulates human intramedullary stage IIIA osteomyelitis of a long bone (Cierny-Mader classification), and the applied foreign-body-associated infection model was aimed to simulate bone cement remnants with *S. epidermidis* biofilms in patients with infected hip arthroplasties. Our aim was to compare the ^18^ F-FDG-PET/CT characteristics of two conditions: acute/subacute osteomyelitis with *S. aureus* versus foreign-body-associated subacute/chronic osteomyelitis with *S. epidermidis*. This study did not serve as a head-to-head comparison of the two staphylococcal strains, because the two models required different treatments (use of sodium morrhuate in *S. epidermidis* animals and the removal of bone cement in *S. aureus* animals).

In the current experiment, CT was successfully applied to anatomically localize the high uptake of ^18^ F-FDG in the medullary cavity of the infected tibias. Still, the rabbit bone is relatively small for human PET/CT scanners with a relatively low spatial resolution, carrying a risk for partial-volume effect [30]. We were not able to apply high-resolution PET/micro-CT/MRI imaging techniques because these scanners are feasible only for smaller animals like mice and rats.

The selection of a single time point for PET imaging (8 weeks after bacterial inoculation) was based on our previous experiments of ^18^ F-FDG PET imaging of *S. aureus* osteomyelitis in the same model [15,17]. The selected time point is clinically relevant to confirm that the acute culture-positive infection detected at 2 weeks was persisting and progressing into a chronic state. It is probable that a longer follow-up would have resulted in healing of some of low-grade *S. epidermidis* infections. A weekly PET scanning might have provided additional information, but the potential risks of repeated general anesthesia were considered to overweigh the expected benefits.

As an additional limitation, we did not perform concurrent evaluation of infections by means of other imaging modalities. For example, MRI could have demonstrated distinct differences between the groups irrespective of ^18^ F-FDG PET results. In the future, evolution of PET/MRI scanners may provide a new way to better characterize bone infections.

## Conclusions

This experimental ^18^ F-FDG PET/CT study demonstrated that the degree of leukocyte infiltration, reflecting the virulence of the causative staphylococcal strain, contributes to the level of local ^18^ F-FDG uptake. SUV values were high in the animals with culture-positive *S. aureus* osteomyelitis. *S. epidermidis* inoculations, independently on the consistency of subsequent culture-positive infections, were characterized by low ^18^ F-FDG uptake.

## Competing interests

The authors declare that they have no competing interests.

## Authors’ contributions

HTA, AR, AJH, and PL designed the study. PL and KL carried out the experimental aspects of the study. PL and HTA drafted the manuscript. All authors read and approved the final manuscript.
